# Autologous conditioned serum (Orthokine) injection for treatment of classical trigeminal neuralgia: results of a single-center case series

**DOI:** 10.1186/s13256-022-03393-9

**Published:** 2022-05-08

**Authors:** Dawood Aghamohammadi, Shahrzad Sharifi, Seyed Kazem Shakouri, Yashar Eslampour, Neda Dolatkhah

**Affiliations:** 1 Palliative Care Medicine Department, Faculty of Medicine, Tabriz University of Medical Sciences, Tabriz, Iran; 2 Physical Medicine and Rehabilitation Research Center, Aging Research Institute, Tabriz University of Medical Science, Tabriz, Iran

**Keywords:** Autologous conditioned serum, Trigeminal neuralgia, Numeric rating scale

## Abstract

**Background:**

Despite some advances, treatment of trigeminal neuralgia remains a significant challenge. This study determines the efficacy and safety of autologous conditioned serum (Orthokine) injection into the foramen oval to treat refractory trigeminal neuralgia.

**Case presentation:**

This is a consecutive case series from the Pain and Palliative Care Department of Imam Reza University Hospital, Tabriz, Iran. Eleven Iranian patients, eligible according to the inclusion and exclusion criteria, aged 45.64 ± 11.58 years (Four male and seven female, all Iranian) with established classical trigeminal neuralgia were injected with Orthokine (2 mL per injection) once a week for three consecutive weeks (total of four injections). Numeric rating scale scores for facial pain intensity and also carbamazepine daily dose were confirmed at pretreatment (T0) and at week 1 (T1), week 2 (T2), week 3 (T3), week 4 (T4), and month 2 (T5) posttreatment. Pain intensity was significantly reduced in the first 3 weeks of follow-up in comparison with baseline (T0 to T3) (8.18 ± 1.99 to 2.82 ± 2.13, *p* < 0.001), an effect that was retained at week 4 (T4) and month 2 (T5) follow-ups (2.82 ± 2.13 to 3.36 ± 2.69, *p* = 0.886). Carbamazepine consumption was significantly reduced in the first 3 weeks of follow-up in comparison with baseline (T0 to T3) (636.36 ± 307.48 to 200.00 ± 296.64, *p* = 0.003), an effect that was retained at week 4 and month 2 follow-ups (200.00 ± 296.64 to 200.00 ± 282.84, *p* = 0.802). There were no serious adverse events in participants.

**Conclusion:**

Orthokine injection led to consistent pain relief and reduced carbamazepine dosage in patients with trigeminal neuralgia, with acceptable safety.

## Background

Trigeminal neuralgia (TGN) is described as repeated hemifacial attacks of highly intense, electrical stun-like or electric pains restricted to the area innervated by the first and/or second and/or third distributions of the trigeminal nerve. It lasts from a few moments to a few minutes and is provoked by apparently innocuous sensory stimulants, including airflow over the face, speaking, chewing, or brushing the teeth [1]. As the disorder progresses, the frequency of pain may increase, significantly increasing patients’ risk of anxiety and depression and significantly impairing their quality of life [2], Hence, pain management is vital for these patients.

The underlying cause and mechanisms of TGN are not well understood and are classified into idiopathic TGN, classical TGN, and secondary TGN according to the cause of TGN [3]. Unidentified origins describe idiopathic TGN, even after surgery or diagnostic imaging, in about 10% of patients [4]. Classic TGN is correlated with neurovascular compression (NVC) in the trigeminal root entry zone (REZ), leading to atrophy or dislocation of the nerve root [4, 5]. Secondary TGN may be triggered by a fundamental disorder such as tumors or blood vessel deformities associated with multiple sclerosis [6].

Despite current progress, TGN treatment remains a significant challenge. The only US Food and Drug Administration-approved first-line treatment is carbamazepine, a voltage-gated sodium-channel blocker [7]. Meanwhile, first-line treatments can alleviate pain in about 80% of patients [8]. However, these treatments are imperfect because of their low tolerability, the need for careful titration, and the possibility of pharmacological interactions, losing efficiency over time [7, 9]. More aggressive interventions are generally reserved for subjects with unsuccessful pharmacological therapy, including microvascular decompression [10], various ablative procedures [11, 12], mechanical balloon compression [13], chemical injection [14, 15], radiosurgery with gamma-knife [16], and peripheral neurectomy. Some of these may lead to multiple complications, for example, microvascular decompression and hearing loss [17]. Therefore, novel and innovative treatment modalities are necessary.

Vascular compression of the fifth or seventh cranial nerve roots and consequently nerve demyelination in the REZ is the principal hypothesis for classic TGN pathophysiology [18]. Once the nerve is injured, Schwann cells and macrophages phagocytize the deteriorated myelin, creating more cytokines that exacerbate demyelination [19–21]. Several recent studies have shown a close association between inflammation and TGN. Some inflammatory factors, such as tumor necrosis factor, chemokine (C–C motif) ligand 2 (CCL2), hemokine (C–C motif) ligand 5, interleukin 6, and transient receptor potential cation channel subfamily A member 1, have been shown to prompt neuropathic pain [22–25] .

Autologous conditioned serum (ACS) is an autologous blood product, where a patient’s peripheral blood is drawn and incubated for hours, throughout which platelets degranulate and mononuclear cells release IL-1 receptor antagonist in addition to a variety of antiinflammatory cytokines and growth factors. The ACS is then retrieved, disinfected, and injected into places of damage or disease [26]. At this time, ACS is advertised under the name Orthokine. Nowadays, ACS can be manufactured locally in a private clinic or physician’s workplace. Though the original 50-mL syringe needed 24-hour incubation at 37 °C for ideal ACS production, the new 10-mL Orthokine syringe enables a shorter incubation time of 6–9 hours. The cost of the injection procedure is determined by the number of syringes and injections applied. Generally, three to six injections are used. Furthermore, the cost may differ depending on the physician.

Over recent decades, the efficacy and safety of ACS for musculoskeletal disorders have been studied in several animal and human investigations, along with *in vivo* and *in vitro* [27–31]. Joint diseases, especially osteoarthritis, are the most common disorders subjected to these investigations, where some evidence mostly indicates that ACS is harmless and effective for pain management and function enhancement. It is also expected to have long-lasting special effects [32]. Based on their clearly developing utilization, medical literature has tended to assess more blood products such as platelet-rich plasma and ACS over the last decade. Another reported use of ACS is its epidural injection for radiculopathy patients [33–35]. Significant improvement was reported in terms of symptom relief. However, these were mostly pilot studies with insufficient sample sizes and provide relatively low-quality evidence.

In recent decades, little progress has been made in management of TGN, and there is a lack of high-quality studies in this area. At present, it is irrefutable that less aggressive and remedial rather than palliative therapeutic alternatives (for example, injectable blood products compared with pain control by oral medicines or surgical procedures) are generally more preferable for both physicians and patients. Considering the close relationship between TGN and inflammation and the evidence of aggregation of a great number of macrophages and mast cells in focal demyelination of the trigeminal root in addition to significant inflammatory infiltrate neighboring the demyelinated areas in TGN animal models [36, 37], we hypothesized that Orthokine would alleviate clinical features in refractory TGN patients. This case series aims to define the clinical efficacy and safety of Orthokine in decreasing pain and drug usage in adult patients with TGN. To the best of the authors’ knowledge, this study is the first to investigate the effect of Orthokine injection in treatment of classical TGN.

## Case series presentation

From July 2019 to March 2020, a total of 23 consecutive patients with medically refractory idiopathic TGN were assessed at the pain and palliative outpatient clinic of Imam Reza University Hospital, Tabriz, Iran, of whom 10 patients did not satisfy the inclusion criteria and 2 rejected participation in the study.

Adults aged 18–70 years with confirmed TGN with at least three pain attacks per day, pain intensity of four or more on a pain intensity numerical rating scale (PI-NRS) on at least 7 days before the start of treatment, history of previous intake of carbamazepine 600–900 mg/day, gabapentin 900–1800 mg/day, or pregabalin 300–450 mg/day and tramadol 400 mg/day for more than 3 months with poor clinical response and a desire to participate throughout the study were included. The criteria for TGN were according to the International Classification of Headache Disorders [5].

Exclusion criteria were previous radiofrequency ablation and/or surgical treatment for TGN, existence of a tumor or autonomic symptoms based on brain magnetic resonance imaging (MRI), or other neuropathic pain, cognitive dysfunction, history of drug abuse, or any coagulation conditions or platelet disorders. Imaging was performed to confirm the lack of a secondary cause.

Finally, 11 Iranian patients (4 male, 7 female) were included in this study. The right and left side was involved in seven and four patients, respectively. The mean age was 45.64  ±  11.58 years (Table 1).

**Table 1:** Demographic characteristics

Characteristic	Value
Age (years), mean ± SD	45.64 ± 11.58
Weight (kg), mean ± SD	76.92 ± 8.97
Female sex, *n* (%)	7 (63.6%)
Right side, *n* (%)	7 (63.6%)
Pain duration (months), mean ± SD	27.09 ± 15.48

*TN* trigeminal neuralgia

The research proposal was approved by the Ethics Committee of the Tabriz University of Medical Sciences on 20/05/2019 (IR.TBZMED.REC.1398.170) in accordance with the Helsinki Declaration of the World Medical Association (version 19 October 2013).

All patients were visited by a palliative care medicine fellow at the study center, who assessed the eligibility criteria, presented necessary information concerning the study, product, alternatives, and risks, and obtained written informed consent. The participants then completed all the baseline questionnaires.

All patients who met the eligibility criteria listed above received diagnostic brain MRI with contrast to rule out any condition or disorder that would deprive them of participation such as compression of Gasser ganglion. Comprehensive demographic information of patients, including age, gender, occupation, and education, was recorded. Medical history concerning consumption of all medications (doses and duration) was obtained.

All patients were accurately evaluated concerning the location, duration, nature, and pain intensity. They were asked to rate their pain on a NRS varying from 0 (“none”) to 10 (“unbearable”).

### Procedure for Orthokine preparation

Fifty milliliters of whole blood samples were taken from each participant using a special syringe (EOT^®^II) [38]. Medical-grade glass beads in the syringes expand the nonpyrogenic surface area. These glass spheres induce the release of IL-1Ra from white blood cells in venous blood samples incubated for 7 hours at 37 °C. The blood-filled syringes were then centrifuged (3000×*g*) for 15 minutes. Before injection, the serum supernatant was filtered and tested for human immunodeficiency virus, syphilis, and hepatitis B and C.

### Procedure for Orthokine injection

Orthokine serum (2 mL per injection) was injected under fluoroscopy direction. All patients had an appointment for an injection once per week for three consecutive weeks (four injections).

**Fig. 1:**
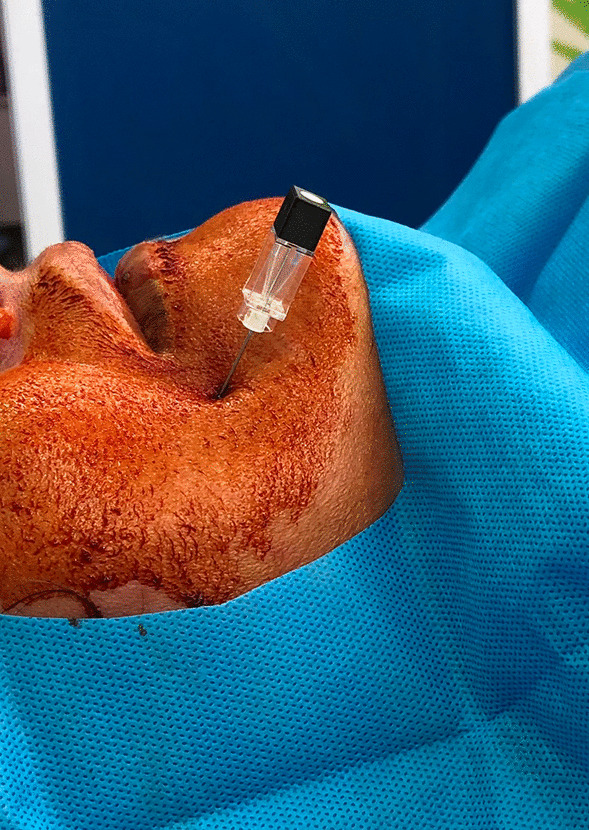
Needle insertion and advancing into the foramen ovale

**Fig. 2:**
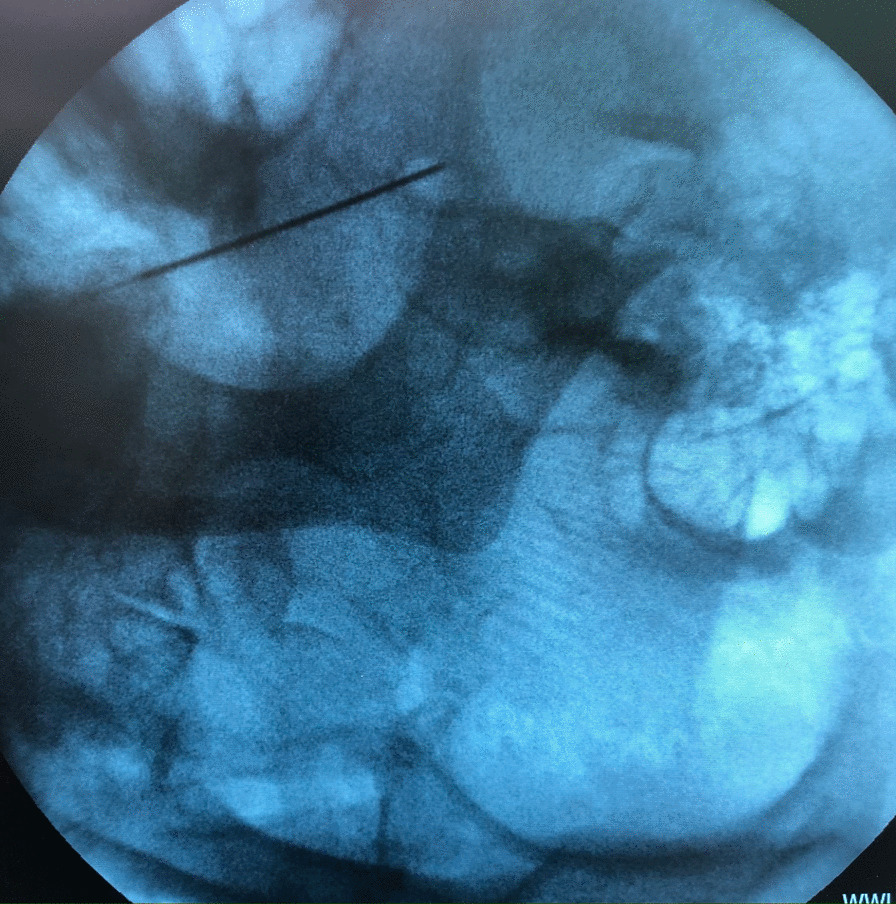
Fluoroscopic image of Orthokine injection

We asked the patient to lie in supine position. Then, a sterile 21-gauge needle with a 10-mm naked needle tip was inserted into the skin 1.5–2 cm lateral to the corner of the mouth, below the lower lip, under fluoroscopy direction in aseptic conditions, and advanced into the foramen ovale (FO), then aspirated. The needle was left in place (Fig. 1). An anesthetic consisting of approximately 2 mL 2% lidocaine was injected to prevent post-injection pain due to the injection itself, then 2 mL Orthokine was injected (Fig. 2). This procedure was the same for each participant. All procedures were done outpatient by one physician, considering all aseptic provisions inside the operation room.

### Patient evaluation and follow-up

Patients who received ≥ 1 dose of Orthokine were asked to document their daily pain intensity, drug usage, and adverse events (AEs) carefully at 1 week before (T0) and 1 month after treatment end (T5). Additionally, the patients were followed up at 1 week (T1), 2 weeks (T2), 3 weeks (T3), and 1 month (T4) after the start of the study, at the pain outpatient clinic.

No drugs were recommended in participants with complete pain relief (NRS < 3). Otherwise, they were counseled to continue carbamazepine up to 400 mg three times a day orally. The same technician executed all follow-up evaluations. The safety profile of ACS was also examined by analysis of AEs at each study visit.

Any AEs, comprising bleeding at the injection site, infection, and other possible AEs, were recorded at weeks 1, 2, 3, and 4, and month 2.

### Statistical methods

Statistical analyses were carried out using SPSS 18.0 software. Continuous variables are presented as mean ± standard deviation, and categorical data as number and percentage. The Shapiro–Wilk test was applied to analyze the distribution. Repeated-measure analysis of variance (ANOVA) testing was applied. *p*-Value < 0.05 was considered significant.

### Pretreatment pain severity

Patients most frequently reported multiple pain attacks daily (eight patients, 72.7%). Two and one patients reported one attack daily and continuous pain, respectively. The mean best and worst pretreatment pain intensity (current 24 hours) was 5.36 ± 2.37 and 10.00 ± 0.00, respectively.

### Efficacy data

**Fig. 3:**
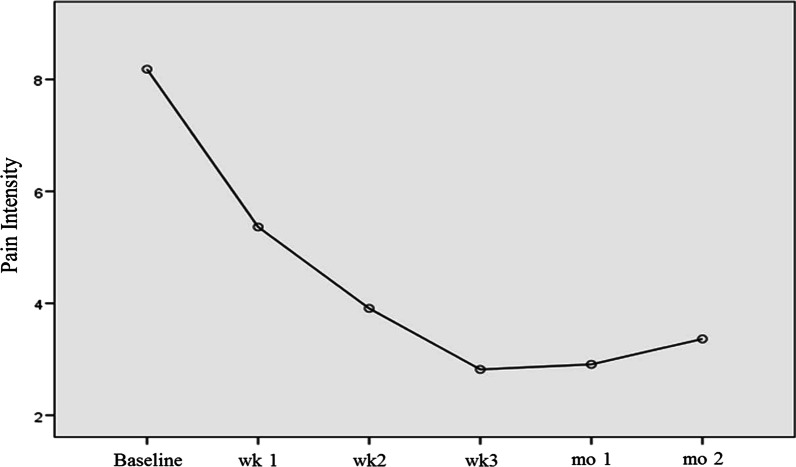
Pain intensity in participants throughout the study

Table 2 presents the pain intensity and carbamazepine consumption in all patients. Orthokine injection led to 31.00 ± 29.33%, 49.31 ± 26.81%, 60.04 ± 32.75%, 56.46 ± 49.69%, and 52.92 ± 49.16% reduction in pain (NRS) at 1 week, 2 weeks, 3 weeks, 4 weeks, and 2 months after beginning treatment. Pain intensity was significantly reduced in the first 3 weeks of follow-up in comparison with baseline (T0 to T3), an effect that was retained at week 4 (T4) and month 2 (T5) follow-ups. Three (27.3%) patients were completely pain free at week 2 follow-up, while two and none of them remained pain free at week 3 and month 1 follow-up, respectively (Fig. 3).

**Table 2:** Pain severity and carbamazepine consumption in all participants

	Pain severity (NRS)						Carbamazepine consumption per day					
	Baseline	1 week	2 weeks	3 weeks	1 month	2 months	Baseline	1 week	2 weeks	3 weeks	1 month	2 months
Mean	8.18	5.36	3.91	2.82	2.91	3.36	636.36	527.27	290.91	200.00	181.82	200.00
SD	1.99	1.91	1.92	2.13	2.46	2.69	307.48	313.34	207.14	296.64	275.02	282.84

*NRS* numeric rating scale; *SD* standard deviation

**Fig. 4:**
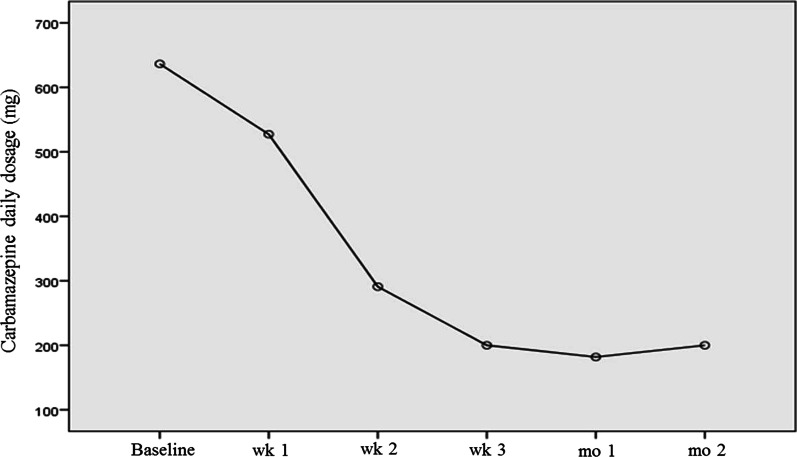
Carbamazepine consumption in participants throughout the study

All patients had used at least one medication for TGN on enrollment in the study. However, they were permitted to continue carbamazepine up to 400 mg three times a day. Carbamazepine consumption was significantly reduced in the first 3 weeks of follow-up compared with baseline (T0 to T3), an effect that was retained at week 4 and month 2 follow-ups (Fig. 4).

### Costs

The overall and per-patient costs were calculated in US $. We considered the costs related to Orthokine as well as the consumables (needle and syringe) needed for its administration. The total cost of Orthokine and consumables per dose administered was US $35.71 and US $1.54, respectively.

### Safety data

Analysis of safety records showed that two transient AEs were reported in five participants: high blood pressure during injection (*n* = 1) and postinjection pain for several days (*n* = 4). There were no serious AEs in participants. No patients were removed from the study because of an AE.

## Discussion

This work reports noteworthy findings regarding pain management of patients with refractory TGN. According to the findings, Orthokine injection into the FO in patients with TGN once a week for three consecutive weeks resulted in a significant reduction in NRS pain intensity, which lasted up to 1 month after the last injection. Also, the dose of carbamazepine in the patients was significantly reduced, an effect that was retained for up to 1 month. Orthokine was safe, and no serious AEs were described in these 11 patients. All seen AEs were transient and remitted with conventional treatment.

Today, there are various concepts and theories about the peripheral and central pathogenesis of TGN. Nerve injury (that is, interstitial neuritis, dystrophy of neural fibers, neural fibers demyelination, and perineural and endoneurial sclerosis) is the pathophysiological mechanism of TGN [39, 40]. Recently, several studies have shown a close association between inflammation and TGN and the role of several inflammatory factors such as IL-1, IL-6, and transient receptor potential cation channel subfamily A member 1 in inducing neuropathic pain [22, 23].

Various strategies have been developed to inhibit the biological activity of IL-1. In particular, IL-1Ra is a naturally occurring IL-1 inhibitor [41, 42]. Macrophages produce this 25-kDa glycoprotein, and certain other types of cells bind to the IL-1 receptor type I without initiating signal transmission, blocking the biological function of IL-1 [43]. The effect of recombinant human IL-1Ra in treating rats with allergic radiculitis has been evaluated in comparison with prednisolone and saline [44]. Based on the findings, both treatments improved the signs and symptoms of experimental polyradiculoneuropathy compared with saline.

Orthokine is rich in antiinflammatory cytokines and has elevated growth factors such as fibroblast growth, hepatocyte growth, and transforming growth factors [45]. It also contains high concentrations of IL-1Ra, a nerve root sensitizer [46]. The mechanism of persistent pain relief in this study may be described by the effect of Orthokine on pain sensitivity or the placebo effect.

Extensive phase III clinical trials have confirmed the safety and efficacy of recombinant IL-1Ra in humans [47]. The efficacy of ACS in musculoskeletal disease has been evaluated and confirmed in several studies [46, 48–50]. However, its use in neuropathic pain has recently attracted attention from researchers in this field. In a double-blind trial, Becker *et al*. [51] evaluated the efficacy of unilateral peripheral neural epidural injection of Orthokine, once a week for three consecutive weeks, compared with triamcinolone in the treatment of 32 patients with lumbar radicular compression, where patients were followed up for up to 6 months. Consistent with our findings, Orthokine resulted in a significant reduction in pain in patients, which was noticeable, clinically significant, and theoretically higher than steroid injection. Kumar *et al*. [35] evaluated the efficacy of perineural epidural injection of Orthokine in 20 patients with unilateral lumbar radiculopathy. Consistent with our findings, they showed a significant reduction in patients’ pain intensity based on the visual analog scale. Orthokine improved the course of the disease and general health simultaneously. Goni *et al*. [52] also evaluated the efficacy of perineural injection of 2.5–3.5 mL Orthokine compared with methylprednisolone in 40 unilateral cervical radiculopathy patients. Patients in both groups showed significant improvement in pain intensity and disability. This improvement was gradual in the Orthokine group and continued throughout the follow-up period, whereas it did not persist in the triamcinolone group. The safety profile of Orthokine was acceptable.

This study has some limitations that should be considered in future studies. This was a single-center single-arm case series study with a small population. Our study did not involve a control group, and the participants could not be masked to the treatment provided. The participants were only monitored for 1 month after treatment. Future long-term follow-up clinical trials across several clinical sites are necessary. This could be executed by directly comparing Orthokine versus arms with other available treatments and/or their combination. We also suggest evaluating other secondary outcomes such as psychiatric symptoms and quality of life.

This study provides significant clinical and therapeutic implications. Though TGN is a rare disorder, it significantly decreases patients’ quality of life because of pain attacks and other related comorbidities, including depression and anxiety. Our study found that patients who underwent Orthokine injection into the FO achieved significantly reduced NRS scores after treatment and significantly lower need for carbamazepine, with no serious AEs, accordingly suggesting the effectiveness of Orthokine treatment. These findings provide solid preliminary evidence that Orthokine is an impressive pain relief technique for refractory TGN.

## Conclusion

The study findings indicate that injection of autologous conditioned serum (Orthokine) into FO led to consistent pain relief and reduced carbamazepine dosage needed for pain suppression in patients with refractory TGN, with acceptable safety. There is a vital necessity for large RCTs to assess the efficacy of Orthokine in TGN conclusively.

## Data Availability

Not applicable.
